# Achromatic linear polarization rotators by tandem twisted nematic liquid crystal cells

**DOI:** 10.1038/s41598-018-32168-w

**Published:** 2018-09-12

**Authors:** Te-Yuan Chung, Min-Cheng Tsai, Cheng-Kai Liu, Jia-Hao Li, Ko-Ting Cheng

**Affiliations:** Department of Optics and Photonics, National Central University, Taoyuan City, 320 Taiwan

## Abstract

An achromatic linear polarization rotator based on a tandem-2ϕ-twisted nematic liquid crystal cell (tandem-2ϕ-TNLC cell, where 2ϕ represents the total twisted angle) is theoretically analyzed and experimentally demonstrated. The tandem-2ϕ-TNLC cell comprises two conventional ϕ-TNLC cells with the required arrangement that the LC director close to the last layer of the first ϕ-TNLC cell should be perpendicular to that close to the first layer of the second ϕ-TNLC cell. With such a simple combination, the TNLC performances are considerably improved. According to the experimental results and theoretical analyses by Jones Calculus, the tandem-2ϕ-TNLC polarization rotator with suitable parameters is achromatic and insensitive to the polarization plane of incident light. Such properties provide these polarization rotators with potential for practical applications.

## Introduction

Controlling the polarization state of light by polarization rotation techniques is important not only in intensity/phase optics but also in several fields of optoelectronics, including display technology, fiber optics, optical communication, and optical measurements^1–5^. In recent decades, devices for controlling the polarization orientation of linearly polarized (LP) lights, including prism rotators (Fresnel rhombs and broadband prismatic rotator), Faraday rotators, and birefringent rotators (half- and quarter-wave retardation plates), have been developed^6^. Among these techniques, the simplest approach to designing a polarization rotator for an LP light that is based on crystal optics is the half-wave retardation plate (λ/2 plate), which can ideally rotate the polarization direction of an LP light with a single wavelength to any other direction^5–7^. However, the wavelength dependence, optical axis orientation of the λ/2 plate, and precise alignment of the optics are inevitable shortcomings of such polarization rotators.

With regard to liquid crystal (LC) polarization rotators, twisted nematic LCs (TNLCs) have been extensively applied in many fields of optics and photonics; these rotators are characterized by their electrical switchability and polarization rotation of LP lights that is based on the waveguide effect^2,3,5,8–11^. The physical mechanism for TNLCs by waveguide effect is based on the superposition of phase retardations from one substrate to the other. The polarization rotation of the output light beam can be simply viewed as its linear polarization rotation with the LC director of the TNLCs. Conventionally, a ϕ-TNLC cell with a twisting degree of ϕ should satisfy the waveguide effect criterion that the output beam must be an LP light; therefore, the Mauguin’s condition (ϕ ≪ 2πdΔn/λ) regarding thickness (d) and the birefringence (Δn) of LCs in a TNLC cell and the wavelength (λ) of the LP incident light should be considered^5,8–11^. Under the Mauguin’s condition, the polarization rotation effect or the waveguide effect results in the polarization rotation of the ϕ degree upon passing through a ϕ-TNLC cell. If the dΔn of the ϕ-TNLC cell is sufficiently large and/or the contained wavelength range of the LP incident light satisfies the Mauguin’s condition, then such a ϕ-TNLC cell is independent of the incident light wavelength. This result confirms that the output light is LP. Furthermore, the limitation of the β angle, which is defined as the angle between the director of the LCs close to the substrate facing the incident light and the polarization direction of the LP incident light, should be 0° or 90° to ensure that the output beam can be LP. Otherwise, the ϕ-TNLC cell transmission under cross- or parallel-polarizers at different β angles will be reduced or increased due to the output elliptically polarized light and the inevitable large rotation angle errors^2,5^. The Gooch–Tarry condition can also be applied to select the TNLC parameters, including the LC thickness (d), LC birefringence (Δn), and wavelength (λ) of the LP incident light, to eliminate the limitation of the β angle^5^. Notably, for practical application of polarization rotation using 90°-TNLCs, satisfying the Gooch–Tarry condition [that is, $\frac{d{\rm{\Delta }}n}{{\rm{\lambda }}}=\sqrt{{m}^{2}-\frac{1}{4}}$ (m is an integer.)] to obtain the property of β angle-independence cannot eliminate the wavelength limitation unless the selected dΔn of the ϕ-TNLC cell or the selected integer (m) is also sufficiently large^2,5^. TNLCs have the major drawbacks of narrow viewing angle, gray scale inversion, and off-axis light leakage from cross-polarizers, but their performances are acceptable for notebook computer applications. Nevertheless, compensated films can be used to enhance the performances of TNLCs.

In addition to the described TNLC polarization rotators, many combined LC polarization rotators and optically controllable polarization rotators have been reported^12–24^. Among them, Komanduri *et al*. introduced broadband LC retarders comprising TNLC layers, which achieve well-controlled polarization transformation for nearly any wavelength, bandwidth, or incident angle range desired^22^. They also identified a method for approaching broadband retarders using multiple twisted birefringent layers on a single substrate. The proposed multi-twist retarders are well suited for patterned achromatic retarders, large bandwidth, and/or low variation of retardation visible through infrared wavelengths^23^. Additionally, Abuleil *et al*. proposed tunable achromatic LC plates that are based on two different nematic LC retarders applied with suitable corresponding voltages^24^.

In the present study, the proposed tandem-2ϕ-TNLCs, where 2ϕ represents the total twisted angle, not only nearly eliminate the indicated limitations but also obtain an LP output light with a high degree of linear polarization (DoLP) and almost zero polarization rotation angle error. The DoLP is defined as (I_max_)/(I_max_ + I_min_), where I_max_ and I_min_ are the measured maximum and minimum intensities, respectively, of the output beam through a rotating linear polarizer^6^. With regard to theoretical analyses, Jones Calculus is utilized to calculate the polarization state of the output beam through the tandem-2ϕ-TNLC cell, which is composed of two ϕ-TNLC cells. The ellipticity and polarization rotation angle of the output polarized light and the tolerance of cell gap differences between the two ϕ-TNLC cells are also discussed. The performances of the tandem-90°-TNLC polarization rotator, composed of two 45°-TNLC cells, are experimentally demonstrated and compared with those of a conventional 90°-TNLC cell. The key requirement for the tandem-2ϕ-TNLC cell is that the LC director of the last layer of the first ϕ-TNLC cell should be perpendicular to that of the first layer of the second ϕ-TNLC cell. According to the reported theoretical analyses and the experimental results, such a tandem-2ϕ-TNLC polarization rotator with suitable parameters is wavelength independent (achromatic) and insensitive to the polarization plane of LP incident lights. Additionally, the polarization rotation angle error, defined as the angle between the expected polarization direction and the major-axis direction of the output elliptically polarized light, can be considerably reduced. The polarization of the output beam through the tandem-2ϕ-TNLC cell is nearly perfect LP light (DoLP = 1). Hence, this proposed polarization rotator exhibits considerable potential for actual applications.

## Theory of Tandem-2ϕ-TNLC Polarization Rotator

A ϕ-TNLC cell with a total twisted angle of ϕ can be considered a stack of equally thin plates of birefringent material with small and consecutive rotation of their optical axes. That is, each thin plate of birefringent material can be viewed as a planar (homogeneous) LC layer. Therefore, the Jones matrix can be completely equal for application to analyze the polarization behavior of a ϕ-TNLC cell with normally incident polarized light. Considering that a ϕ-TNLC cell presents an optical axis on the ***x*** − ***y*** plane, the Jones matrix of such a cell (*M*) can be written as follows^2,5^:

1 $$M=[\begin{array}{cc}\cos \,\varphi  & -\sin \,\varphi \\ \sin \,\varphi  & \cos \,\varphi \end{array}]\,[\begin{array}{cc}\cos \,X-i\frac{{\rm{\Gamma }}}{2}\frac{\sin \,X}{X} & \varphi \frac{\sin \,X}{X}\\ -\varphi \frac{\sin \,X}{X} & \cos \,X+i\frac{{\rm{\Gamma }}}{2}\frac{\sin \,X}{X}\end{array}],$$

where

2 $$\Gamma =\frac{2\pi d{\rm{\Delta }}n}{\lambda },$$

3 $$X=\sqrt{{\varphi }^{2}+{(\frac{\Gamma }{2})}^{2}}.$$

In Eqs (2 and 3), *Γ*, *d*, *Δn*, and *λ* are the phase retardation, thickness of the TNLC cell, LC birefringence, and wavelength of the incident light, respectively. These results hold for an infinite number of LC layers and are generally applicable for a TNLC cell. When the cause of the β angle for the transmittance of a normally white mode transmissive 90°-TNLC cell is considered, the corresponding Jones matrix of a ϕ-TNLC cell between cross-polarizers (M_⊥_) can be expressed as follows:

4 $${M}_{\perp }=[\begin{array}{cc}cos\beta  & sin\beta \end{array}][\begin{array}{cc}\cos \,\varphi  & -\sin \,\varphi \\ \sin \,\varphi  & \cos \,\varphi \end{array}]\,[\begin{array}{cc}\cos \,X-i\frac{{\rm{\Gamma }}}{2}\frac{\sin \,X}{X} & \varphi \frac{\sin \,X}{X}\\ -\varphi \frac{\sin \,X}{X} & \cos \,X+i\frac{{\rm{\Gamma }}}{2}\frac{\sin \,X}{X}\end{array}]\,[\begin{array}{c}-sin\beta \\ cos\beta \end{array}].$$

According to a simple algebraic calculation, the analytical expression for the transmittance (T_⊥_ = |M_⊥_|^2^) is derived as the following equation:

5 $${T}_{\perp }={|{M}_{\perp }|}^{2}={(\frac{\varphi }{X}cos\varphi sinX-sin\varphi cosX)}^{2}+{(\frac{{\rm{\Gamma }}\sin {\rm{X}}}{2{\rm{X}}})}^{2}si{n}^{2}(\varphi -2\beta ).$$

**Figure 1:**
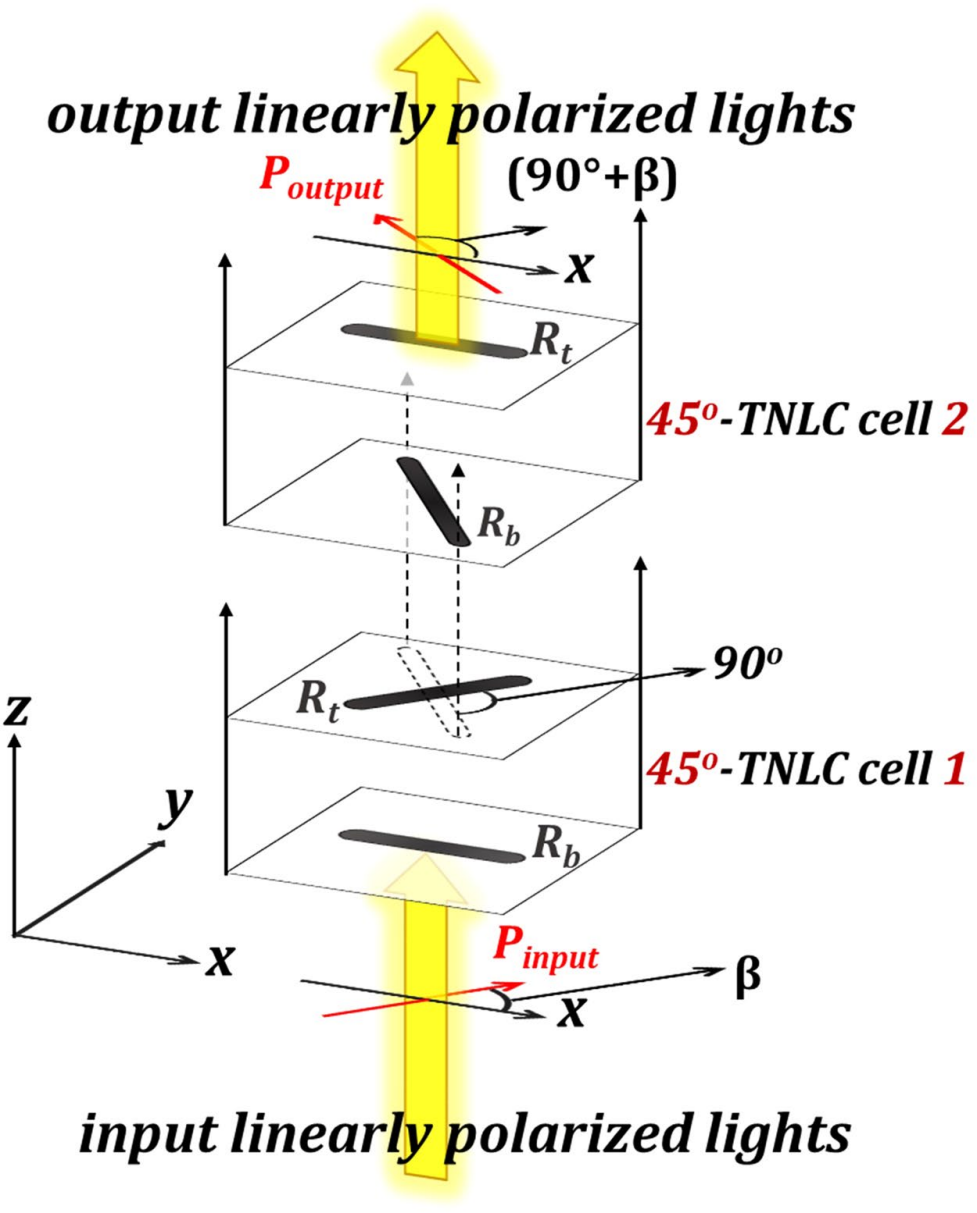
Schematic diagram of the arrangement of the tandem-90°-twisted nematic liquid crystal (TNLC) polarization rotator.

The proposed tandem-2ϕ-TNLC polarization rotator requires a specific orientation of the two identical ϕ-TNLC cells. These cells should be placed in tandem, but the LC director close to the last layer of the first ϕ-TNLC cell should be perpendicular to that close to the first layer of the second ϕ-TNLC cell. Figure 1 illustrates the arrangement of the tandem-90°-TNLC polarization rotator, which consists of two 45°-TNLC cells. *P*_*input*_, *P*_*output*_, *R*_*b*_, and *R*_*t*_ represent the polarization of the input beam, polarization of the output beam, rubbing direction of the bottom substrate, and rubbing direction of the top substrate, respectively. The linear polarization of the input beam can be rotated by 90°, that is, from β to (90° + β), after it passes through the tandem-90°-TNLC polarization rotator. Evidently, the dependence of the β angle based on the tandem-2ϕ-TNLC cells with suitable parameters can be eliminated by self-compensation of undesirable phase retardation, which results from the first ϕ-TNLC cell, using the second ϕ-TNLC cell with such arrangements (Fig. 1).

Hence, the system Jones matrix (*M*_*sys*_) can be written as follows:

6 $${M}_{sys}=[R(-\varphi -\frac{\pi }{2})][M][R(\varphi +\frac{\pi }{2})][M],$$

where *R(θ)* and *M* represent the 2D rotation matrix and the Jones matrix, respectively, of the two ϕ-TNLC cells. The system Jones matrix can then be rewritten as follows:

7 $$\begin{array}{ccc}{M}_{sys}=[{M}_{2}][{M}_{1}] & = & [\begin{array}{cc}-\sin \,\varphi  & -\cos \,\varphi \\ \cos \,\varphi  & -\sin \,\varphi \end{array}]\,[\begin{array}{cc}\cos \,\varphi  & -\sin \,\varphi \\ \sin \,\varphi  & \cos \,\varphi \end{array}]\\  &  & [\begin{array}{cc}\cos \,X-i\frac{{\rm{\Gamma }}}{2}\frac{\sin \,X}{X} & \varphi \frac{\sin \,X}{X}\\ -\varphi \frac{\sin \,X}{X} & \cos \,X+i\frac{{\rm{\Gamma }}}{2}\frac{\sin \,X}{X}\end{array}]\\  &  & [\begin{array}{cc}-\sin \,\varphi  & \cos \,\varphi \\ -\cos \,\varphi  & -\sin \,\varphi \end{array}]\,[\begin{array}{cc}\cos \,\varphi  & -\sin \,\varphi \\ \sin \,\varphi  & \cos \,\varphi \end{array}]\\  &  & [\begin{array}{cc}\cos \,X-i\frac{{\rm{\Gamma }}}{2}\frac{\sin \,X}{X} & \varphi \frac{\sin \,X}{X}\\ -\varphi \frac{\sin \,X}{X} & \cos \,X+i\frac{{\rm{\Gamma }}}{2}\frac{\sin \,X}{X}\end{array}].\end{array}$$

Equation (7) can be further reduced into the following:

8 $$\begin{array}{c}{M}_{sys}=[\begin{array}{cc}-\sin \,2\varphi  & -\cos \,2\varphi \\ \cos \,2\varphi  & -\sin \,2\varphi \end{array}]\\ \,\,\,\,\,\,[\begin{array}{cc}\frac{\varphi \,\sin \,X[-2X\,\cos \,X+i{\rm{\Gamma }}\,\sin \,X]}{{X}^{2}} & co{s}^{2}X+\frac{{{\rm{\Gamma }}}^{2}si{n}^{2}X}{4{X}^{2}}-\frac{{\varphi }^{2}si{n}^{2}X}{{X}^{2}}\\ -co{s}^{2}X-\frac{{{\rm{\Gamma }}}^{2}si{n}^{2}X}{4{X}^{2}}+\frac{{\varphi }^{2}si{n}^{2}X}{{X}^{2}} & \frac{-\varphi [X\,\sin (2X)+i{\rm{\Gamma }}si{n}^{2}X]}{{X}^{2}}\end{array}].\end{array}$$

The following discussion pertains to cases with and without the Mauguin’s condition. First, under the low-twist/large-birefringence limit or the so-called the Mauguin’s condition (X ≈ Γ ≫ ϕ), Eq. (8) can be further simplified into the following:

9 $${M}_{sys}=[\begin{array}{cc}\cos \,2\varphi  & -\sin \,2\varphi \\ \sin \,2\varphi  & \cos \,2\varphi \end{array}]=[{\rm{R}}(-\,2\varphi )].$$

According to the theoretical calculation and approximation, Eq. (9) represents that such a tandem-2ϕ-TNLC cell can be an ideal linear polarization rotator without the dependencies of the wavelength and the polarization plane of the incident lights. The output beam polarization is confirmed as LP with a polarization rotation of exactly 2ϕ with respect to its incident polarization direction. If the approximation of the Mauguin’s condition is not obtained, then Eq. (8) can be rewritten as follows:

10 $$\begin{array}{c}{M}_{sys}=[\begin{array}{cc}-\sin \,2\varphi  & -\cos \,2\varphi \\ \cos \,2\varphi  & -\sin \,2\varphi \end{array}]\,[\begin{array}{cc}0 & 1\\ -1 & 0\end{array}]\,[\begin{array}{cc}{A}_{11} & {A}_{12}\\ {A}_{21} & {A}_{22}\end{array}]\\ \,\,\,\,\,\,=[\begin{array}{cc}\cos \,2\varphi  & -\sin \,2\varphi \\ \sin \,2\varphi  & \cos \,2\varphi \end{array}]\,[A]\\ \,\,\,\,\,\,=[{\rm{R}}(-2\varphi )][A],\end{array}$$

where matrix [*A*] is the following:

11 $$[A]=[\begin{array}{cc}co{s}^{2}X+\frac{{{\rm{\Gamma }}}^{2}si{n}^{2}X}{4{X}^{2}}-\frac{{\varphi }^{2}si{n}^{2}X}{{X}^{2}} & \frac{\varphi [X\,\sin (2X)+i{\rm{\Gamma }}si{n}^{2}X]}{{X}^{2}}\\ \frac{\varphi \,\sin \,X[-\,2X\,\cos \,X+i{\rm{\Gamma }}\,\sin \,X]}{{X}^{2}} & co{s}^{2}X+\frac{{{\rm{\Gamma }}}^{2}si{n}^{2}X}{4{X}^{2}}-\frac{{\varphi }^{2}si{n}^{2}X}{{X}^{2}}\end{array}].$$

Therefore, matrix [A] dominates the polarization state of the output beam through the tandem-2ϕ-TNLC cell. Angle Ω is introduced to facilitate further reduction, thereby obtaining the following:

12 $$\frac{{\rm{\Gamma }}}{2X}=\,\sin \,{\rm{\Omega }},$$

13 $$\frac{\varphi }{X}=\,\cos \,{\rm{\Omega }}.$$

Notably, Ω ranges between 0 and π/2, that is, 0 ≤ Ω ≤ π/2, and Eq. (11) is transformed as follows:

14 $$[A]=[\begin{array}{cc}co{s}^{2}X+si{n}^{2}X(si{n}^{2}\,{\rm{\Omega }}-co{s}^{2}\,{\rm{\Omega }}) & cos{\rm{\Omega }}[\sin (2X)+2i\,sin\,{\rm{\Omega }}\,si{n}^{2}X]\\ 2cos\,{\rm{\Omega }}\,sin\,X(\,-\,cos\,X+i\,sin\,{\rm{\Omega }}\,sin\,X) & co{s}^{2}X+si{n}^{2}X(si{n}^{2}\,{\rm{\Omega }}-co{s}^{2}\,{\rm{\Omega }})\end{array}].$$

With Eq. (14), the optical properties of such a tandem-2ϕ-TNLC cell can be evaluated. The effects of the β angle of the tandem-2ϕ-TNLC cell on the output beam polarization are discussed in the Results and Discussion Section.

## Results and Discussion

### Theoretical calculations

**Figure 2:**
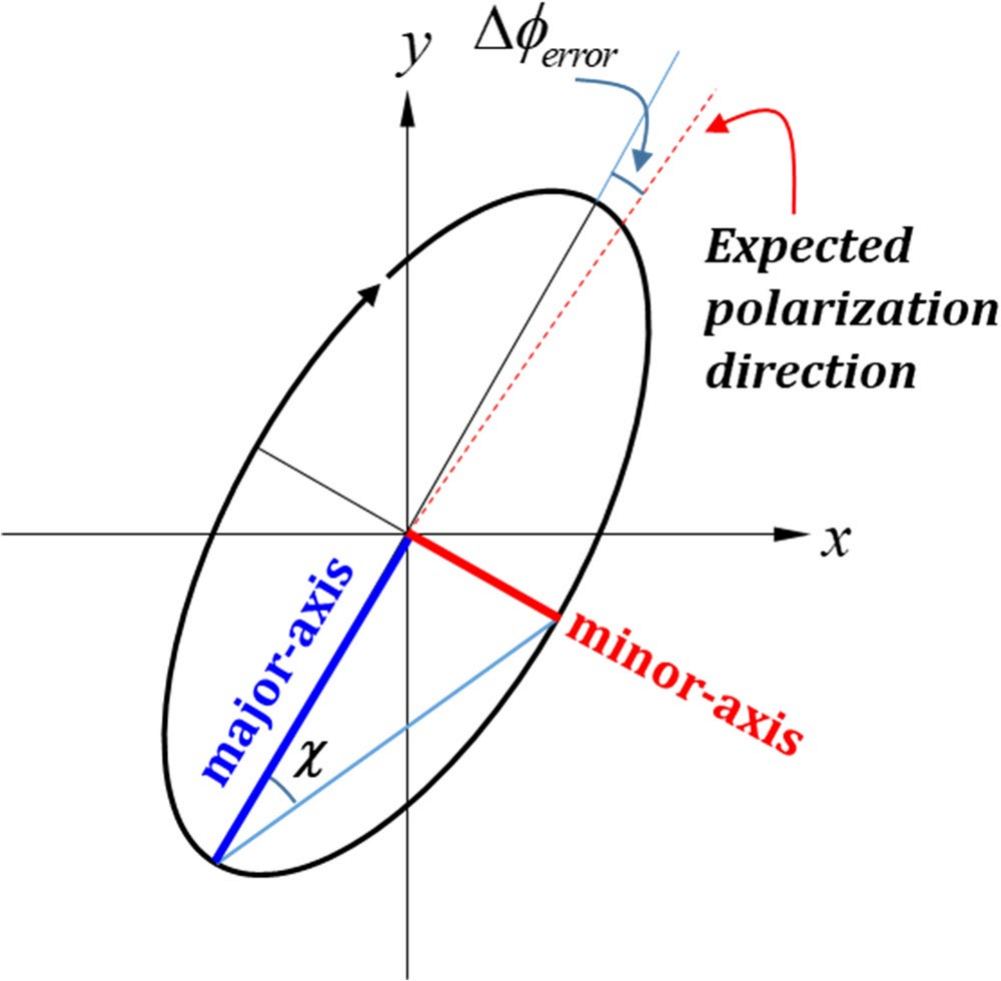
Definitions of two parameters, χ and Δϕ_error_ angles, for describing elliptically polarized light.

**Figure 3:**
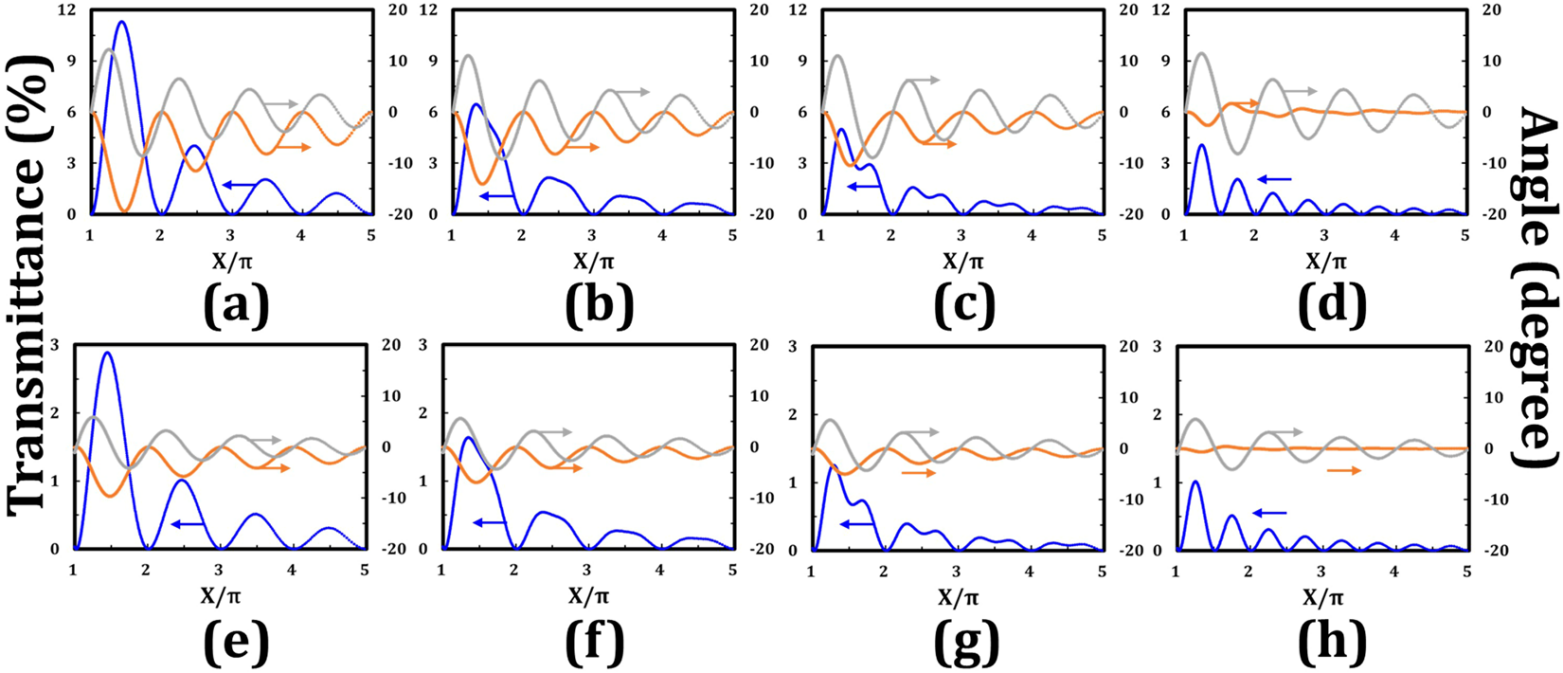
Theoretical minimum transmittance (blue) of the output beam through a tandem-2ϕ-TNLC cell and an analyzer with its transmissive axis perpendicular to the major-axis of the output polarized light, χ angle (orange), and Δϕ_error_ angle (gray) of the output polarized beam through a tandem-2ϕ-TNLC cell versus the X/π values at different β angles with ϕ of (**a**–**d**) 45° and (**e**–**h**) 22.5°, as calculated by Jones Calculus. (**a**,**e**), (**b**,**f**), (**c**,**g**) and (**d**,**h**) figures represent the different β angles of 0°, 22.5°, 30°, and 45°, respectively. The titles of the primary and the secondary vertical axes for the eight figures are transmittance (%) and angle (degree), respectively.

The effect of the β angle in the tandem-2ϕ-TNLC cell on the minimum transmittance of the output beam passing through a linear polarizer, polarization state, and polarization rotation angle of the output beam according to tandem-2ϕ-TNLC polarization rotator theory are discussed in this section. The minimum transmittance (T_min_) is defined as the transmittance of the output beam through a tandem-2ϕ-TNLC cell and an analyzer with its transmissive axis perpendicular to the major-axis of the output elliptically polarized light. Moreover, two parameters, χ and Δϕ_error_ angles, are introduced to describe the output elliptically and/or LP light in the following discussion. χ is the arctan value of the ratio of the minor-axis and major-axis electric fields. Δϕ_error_ is defined as the polarization rotation angle error, which refers to the angle between the expected polarization direction and the major-axis direction of the output elliptically polarized light, as shown in Fig. 2. First, if the tandem-2ϕ-TNLC polarization rotator and the wavelength of the LP incident light satisfy the Mauguin’s condition (X ≈ Γ ≫ ϕ), then such a polarization rotator is a veritably ideal linear polarization rotator. Therefore, the polarization of the output beam of the LP incident light is the exact linear polarization with polarization rotation angle of 2ϕ, as depicted in Eq. (9). In the absence of the approximation of the Mauguin’s condition, according to Eq. (14), the theoretical T_min_, χ, and Δϕ_error_ angles of the output beam through a tandem-2ϕ-TNLC cell versus the X/π values of the two cases, namely, ϕ values of 45° (Fig. 3(a–d)) and 22.5° (Fig. 3(e–h)), can be obtained for different β angles (0°, 22.5°, 30°, and 45°). The X value is a function of twisted angle (ϕ), wavelength (λ), birefringence (Δn), and thickness (d) of the LC film. Small ϕ and large X generally yield small T_min_, χ, and |Δϕ_error_| angles according to the theoretical results. This finding can be attributed to that the conditions imply that the polarization rotator satisfies the Mauguin’s condition. According to the theoretical analyses, the calculated T_min_, χ, and |Δϕ_error_| angles of the case of ϕ value of 22.5° (Fig. 3(e–h)) are better than those of ϕ value of 45° (Fig. 3(a–d)) because the combined parameters of the 22.5° case are more consistent with the Mauguin’s condition than those of the 45° case.

According to the calculated results, several notable features can be observed when the X/π value is an integer or half of an integer. According to Eq. (14), when X = nπ (n ∈ natural number), the sinX value equals to 0 and the matrix [A] becomes an identity matrix, that is, $$[\begin{array}{cc}1 & 0\\ 0 & 1\end{array}]$$. Hence, the DoLP value of the output light remains unchanged (DoLP = 1), and an ideal polarization rotation of 2ϕ exists after penetrating through the tandem-2ϕ-TNLC cell. The condition X = nπ is also known as the Gooch–Terry condition^2,5^.

In addition, when the X value equals (2n + 1)π/2 (n ∈ natural number), the matrix [A] can be reduced as follows:

15 $${[A]|}_{X=(2{\rm{n}}+1){\rm{\pi }}/2}=[\begin{array}{cc}-cos\,2{\rm{\Omega }} & i\,sin\,2{\rm{\Omega }}\\ i\,sin\,2{\rm{\Omega }} & -cos\,2{\rm{\Omega }}\end{array}].$$

The eigenvectors of this matrix, that is, [*A*]|_*X*=(2n+1)π/2_, are $$\frac{1}{\sqrt{2}}[\begin{array}{c}1\\ 1\end{array}]$$ and $$\frac{1}{\sqrt{2}}[\begin{array}{c}1\\ -\,1\end{array}]$$, which are +45° and −45° LP lights, respectively. These eigenvectors also indicate that the output lights of the two incident polarization states are also ideal LP lights and obtain a polarization rotation of 2ϕ with respect to their incident polarization directions.

**Figure 4:**
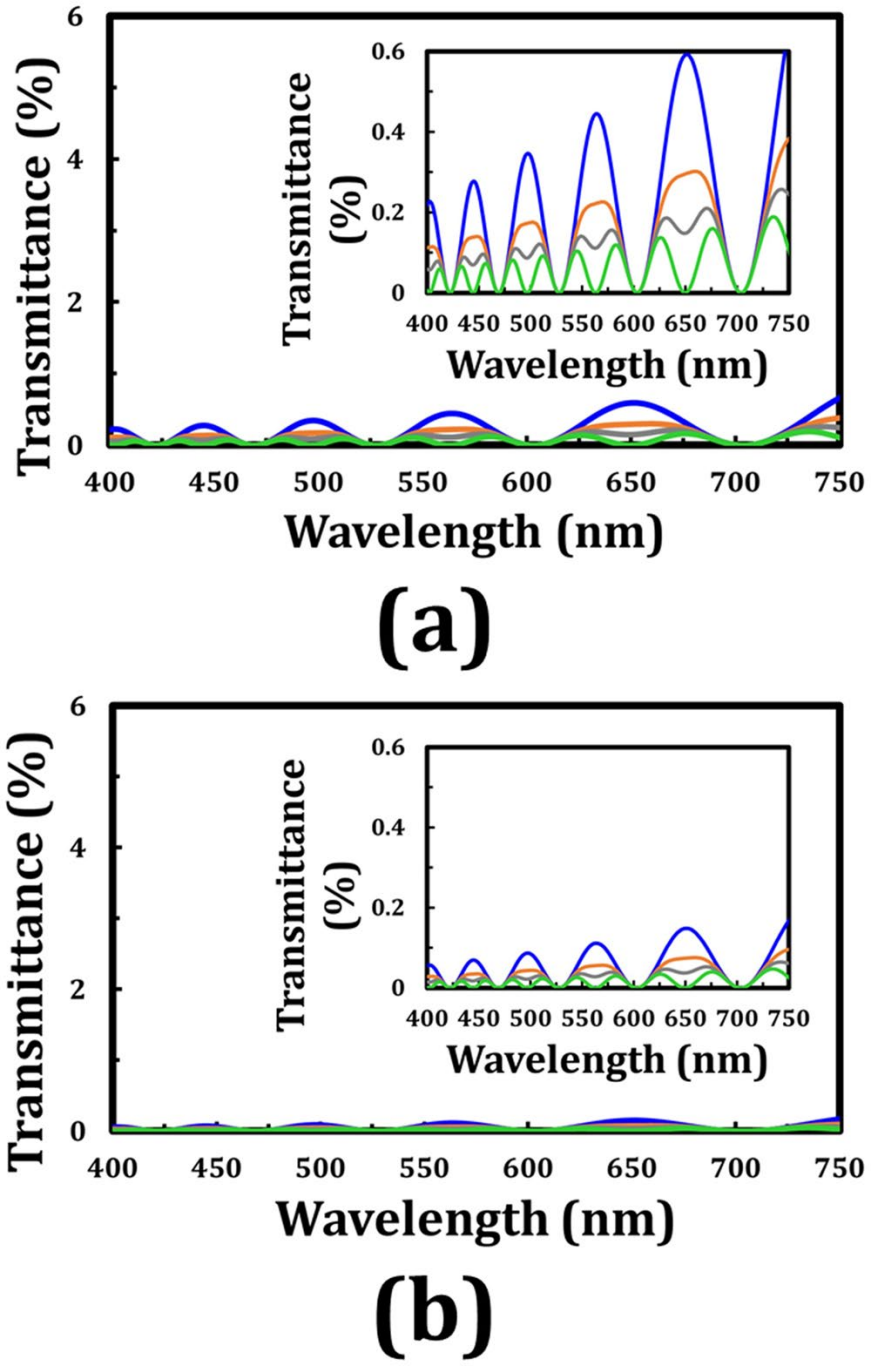
Theoretical transmittance (Jones Calculus) of the output beam through a tandem-2ϕ-TNLC cell and an analyzer with its transmissive axis of 90° + 2ϕ with respect to that of the polarizer (β) versus the wavelength of the input beam at different β angles with ϕ of (**a**) 45° and (**b**) 22.5°. Blue, orange, gray, and green curves represent the different β angles of 0°, 22.5°, 30°, and 45°, respectively. Insets evidently show the details of the spectra. The refractive index dispersion of the adopted LC (E7) for these theoretical analyses is not considered.

With regard to the theoretical calculation, the cell gap of each TNLC cell is 20 μm, and the ordinary and extraordinary refractive indexes and birefringence of the adopted LCs are 1.519, 1.73, and 0.211, respectively. The refractive index dispersion is not considered in this part. Concerning the applications of TNLC cell that are based on normally black mode, the transmittance spectra in the visible range of such tandem-90°-TNLC and tandem-45°-TNLC polarization rotators under the corresponding polarizer–analyzer conditions at β angles of 0°, 22.5°, 30°, and 45° in cases of twisted angles (ϕ) of 45° and 22.5° are correspondingly shown in Fig. 4(a,b). The polarizer–analyzer conditions required to obtain these transmittances indicate that the transmissive axis of the analyzer is set as 90° + 2ϕ with respect to that of the polarizer (β). The transmittance spectra obtained by Jones Calculus for the conditions of β angles of 0° and 90° are consistent with each other (data are not shown). The transmittance is less than 0.6% on average. Theoretically, the wavelength-dependent oscillations of transmittance, whose amplitudes increase with the wavelength, result from the polarization states and the tiny polarization rotation errors of the output polarized light. In the same β angle, the case with ϕ of 22.5° results in approximately another fourfold improvement. This result can be attributed to the fact that the latter case (Fig. 4(b)) satisfies the criterion of the Mauguin’s condition (X ≈ Γ ≫ ϕ) more than does the former one (Fig. 4(a)). In the case of β = 45°, the average transmittance obtains another fourfold improvement compared with that of β = 0° case according to the theoretical analyses shown in Eq. (15). However, if the two TNLC cells satisfy the Mauguin’s condition, then the difference between the two cases and the several tiny wavelength-dependent oscillations of transmittance will be reduced, as presented in the theoretical analyses depicted in Fig. 3. Therefore, the tandem-2ϕ-TNLC with suitable parameters performs well in the polarization rotator on the basis of the self-compensation of the undesirable phase retardation by the two ϕ-TNLC cells (Fig. 1).

**Figure 5:**
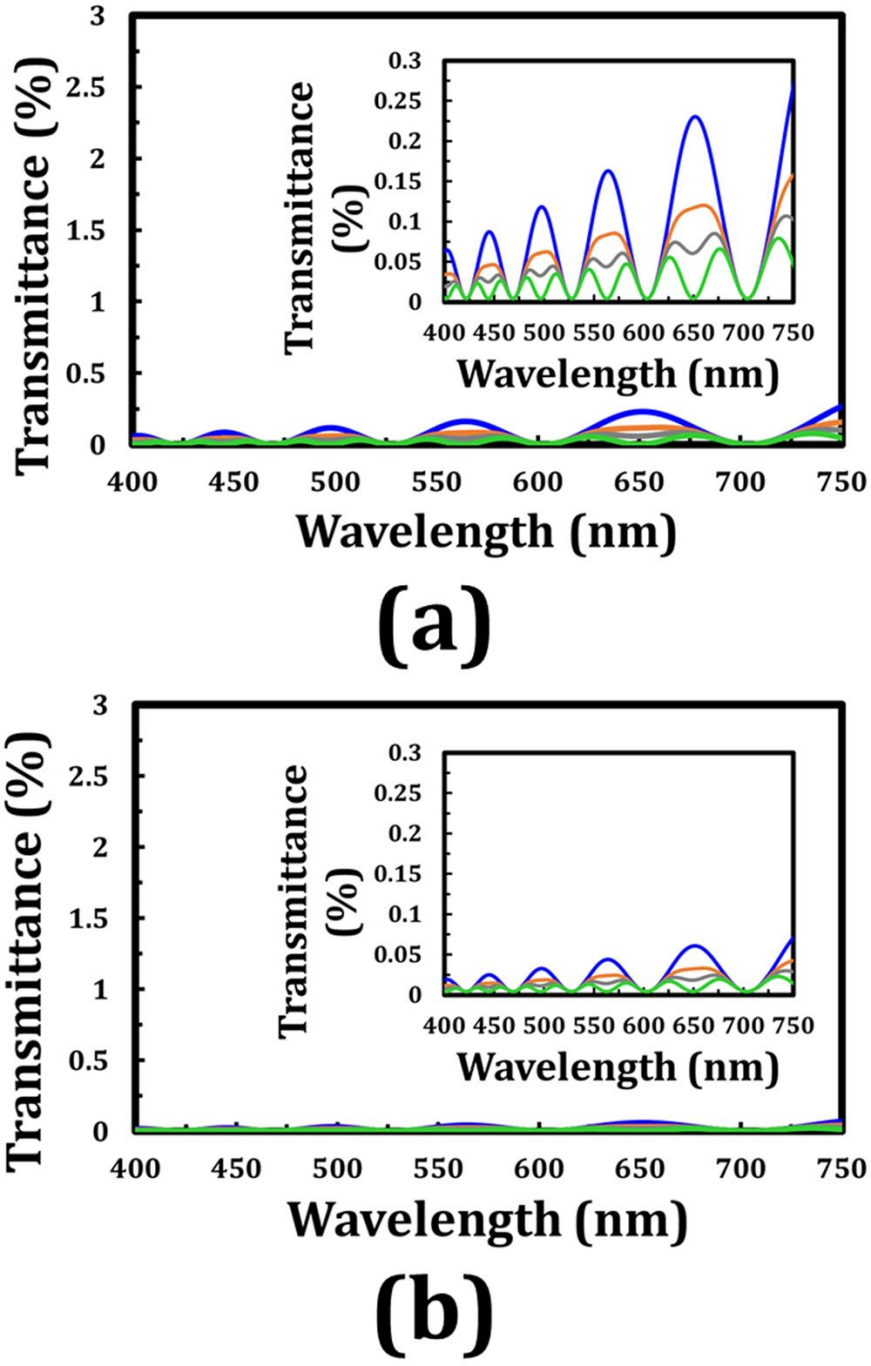
Simulated transmittance (1D-DIMOS) of the output beam through a tandem-2ϕ-TNLC cell and an analyzer with its transmissive axis of 90° + 2ϕ with respect to that of the polarizer (β) versus the wavelength of the input beam at different β angles with ϕ of (**a**) 45° and (**b**) 22.5°. Blue, orange, gray, and green curves represent the different β angles of 0°, 22.5°, 30°, and 45°, respectively. Insets evidently show the details of the spectra. The refractive index dispersion of the adopted LC (E7) for these theoretical analyses is not considered.

In addition to the use of Jones Calculus in validating the performance of the tandem-2ϕ-TNLC polarization rotator, commercial software 1D-DIMOS confirms the concept. The parameters of the LC cells and the adopted LCs and the polarizer–analyzer conditions are consistent with the aforementioned. The refractive index dispersion is also not considered in this part. With different β angles of 0°, 22.5°, 30°, and 45° and ϕ values of 45° and 22.5°, the simulated transmittances versus wavelengths of the two cases can be obtained, as shown in Fig. 5(a,b), respectively. The transmittance spectra obtained by the 1D-DIMOS software for β angles of 0° and 90° are also the same (data not shown). Comparison of the theoretical transmittance spectra calculated by Jones Calculus with the ones simulated through the 1D-DIMOS software evidently show that the performances of the linear polarization rotators are considerably high despite the several small wavelength-dependent oscillations of transmittance increasing with the wavelength, as shown in these curves. The causes of these oscillations based on the polarization rotation angle errors, the Gooch–Terry condition, and the Mauguin’s condition have been discussed in the previous paragraphs. Furthermore, the transmittance shown in Fig. 5 (1D-DIMOS) is slightly lower than that shown in Fig. 4 (Jones Calculus). These results can be due to the ignored light absorbance and reflectance from the linear polarizers and several boundaries in Jones Calculus.

**Figure 6:**
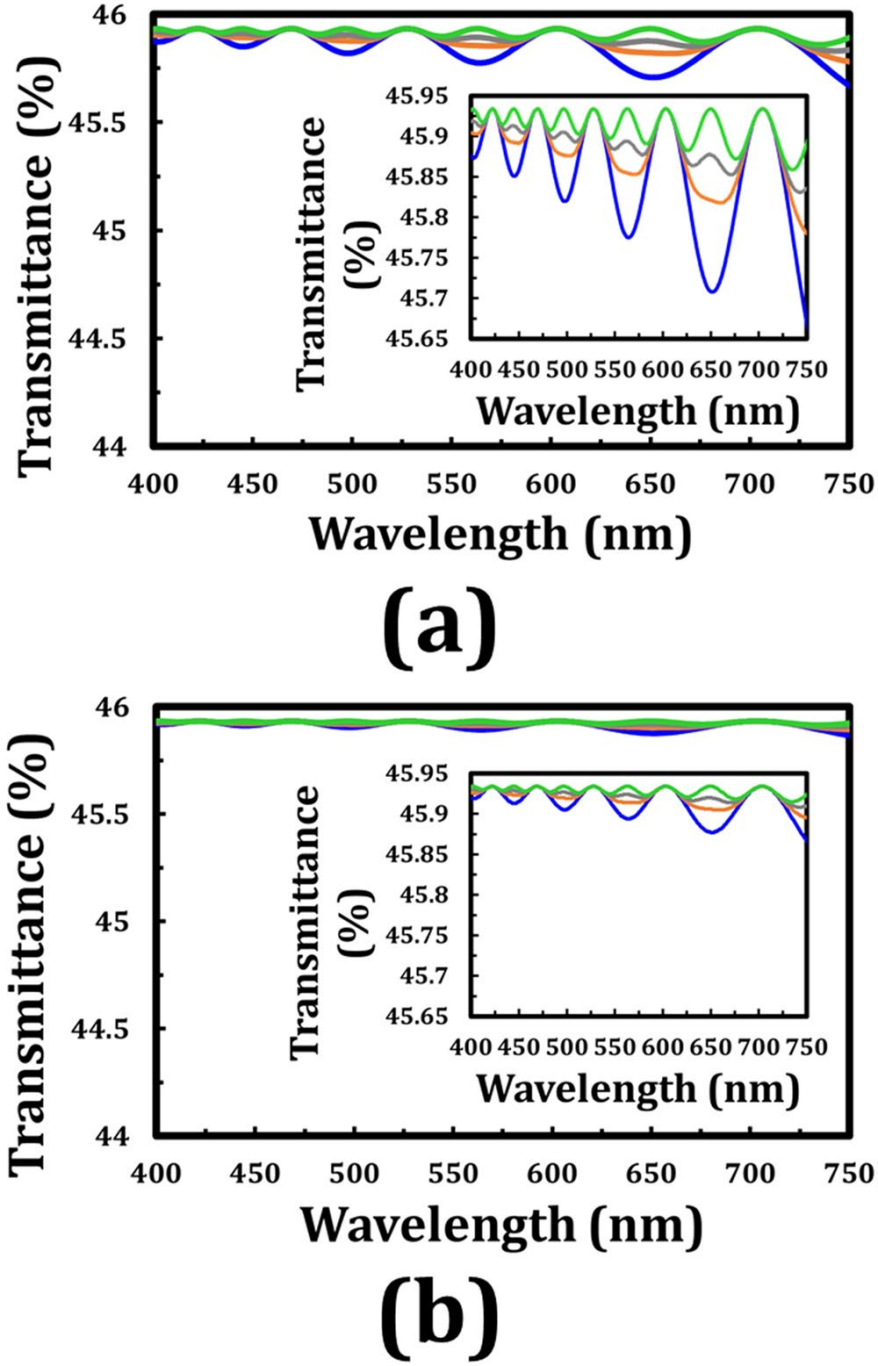
Simulated transmittance (1D-DIMOS) of the output beam through a tandem-2ϕ-TNLC cell and an analyzer with its transmissive axis of 2ϕ with respect to that of the polarizer (β) versus the wavelength of the input beam at different β angles with ϕ of (**a**) 45° and (**b**) 22.5°. Blue, orange, gray, and green curves represent the different β angles of 0°, 22.5°, 30°, and 45°, respectively. Insets clearly show the details of the spectra. The refractive index dispersion of the adopted LC (E7) for these theoretical analyses is not considered.

By contrast, if the polarizer–analyzer conditions are changed to that the transmissive axis of the analyzer is set to 2ϕ with respect to that of the polarizer (β), then the high transmittance of the light passing through the tandem-2ϕ-TNLC linear polarization rotators can be obtained. Figure 6(a,b) show the simulated transmittances of the output beam through the tandem-90°-TNLC and tandem-45°-TNLC cells, respectively, versus the wavelength of the input beam at different β angles. The maximum transmission shown in Fig. 6 is approximately 0.45934, which is lower than the theoretical value of 0.5. The difference can be attributed to the light absorbance and reflectance from the polarizers and several boundaries, and the total transmission of 1 of the unpolarized incident light. Moreover, according to the simulated results, the polarization rotation angle error (Δϕ_error_) of the output LP light through the two kinds of tandem-2ϕ-TNLC is small. Without consideration of the small Δϕ_error_, all the simulated DoLP values of the output lights in the present wavelength range at different β angles through the two kinds of tandem-2ϕ-TNLC linear polarization rotators (ϕ: 22.5° and 45°) are higher than 0.995. Therefore, the output light is nearly an ideal LP light with expected polarization rotation angle (2ϕ).

**Figure 7:**
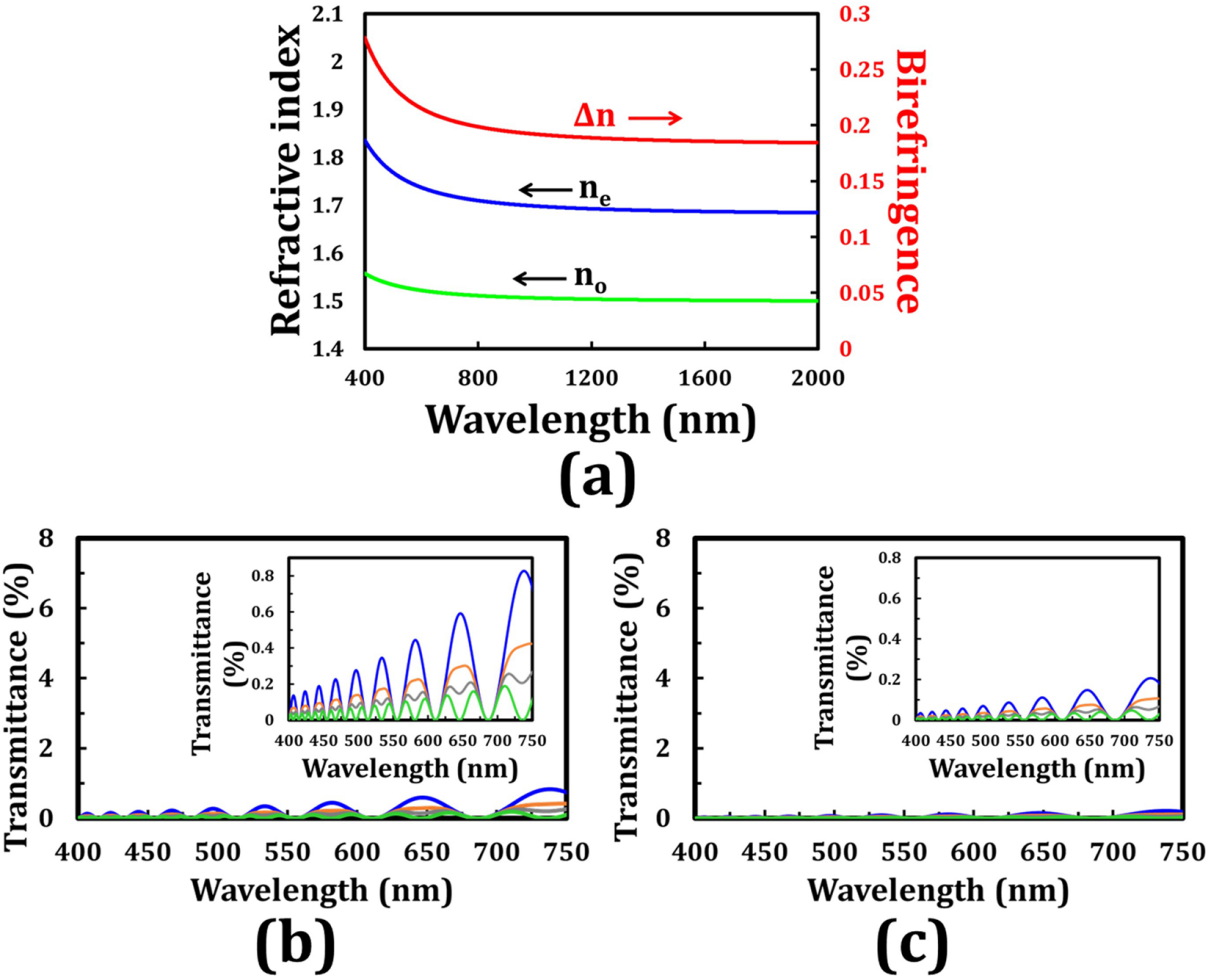
(**a**) Refractive index dispersion of the adopted LC (E7) based on Cauchy formula, where red, blue, and green curves represent the Δn, n_e_, and n_o_, respectively. Theoretical transmittance (Jones Calculus) of the output beam through a tandem-2ϕ-TNLC cell and an analyzer with its transmissive axis of 90° + 2ϕ with respect to that of the polarizer (β) versus the wavelength of the input beam at different β angles with ϕ of (**b**) 45° and (**c**) 22.5°. Blue, orange, gray, and green curves represent the different β angles of 0°, 22.5°, 30°, and 45°, respectively. Insets evidently show the details of the spectra. The refractive index dispersion of the adopted LC (E7) for these theoretical analyses is considered.

The refractive index dispersion of the adopted LC (E7) for the preceding theoretical analyses shown in Figs 4–6 is not considered. Practically, the refractive indexes of LC, including n_e_ and n_o_, depend on the wavelength of the incident light^25,26^. The theoretical transmittance spectra of the output beam through a tandem-2ϕ-TNLC cell and an analyzer with its transmissive axis of 90° + 2ϕ with respect to that of the polarizer (β) based on Jones Calculus and Cauchy formula are obtained to elucidate the important refractive index dispersion effect onto the tandem-2ϕ-TNLC devices. Figure 7(a) plots the refractive index dispersion, including Δn, n_e_, and n_o_, of the adopted LC (E7) on the basis of the Cauchy formula. Figure 7(b,c) show the simulated transmittances of the output beam through the tandem-90°-TNLC and tandem-45°-TNLC cells, respectively, versus the wavelength of the input beam at different β angles. All parameters, except for the refractive index of LC, are consistent with those of Fig. 4. A comparison of Figs 4 and 7 shows that the light leakage and the number of oscillations in the wavelength range (the Gooch–Terry condition) plotted in Fig. 7 are larger than those depicted in Fig. 4. It can be easily understood because the refractive index of the adopted LC decreases with the wavelength of the incident light according to the Cauchy formula (Fig. 3). Moreover, as mentioned in Fig. 4, the theoretical light leakage of the tandem-45°-TNLC cell (Fig. 4(b)) is lower than that of the tandem-90°-TNLC cell (Fig. 4(a)) due to the satisfaction of the Mauguin’s condition. With the increase in the cell gap, the difference between the two cases will be reduced, as depicted in Fig. 3.

Additionally, the polarization rotation angle errors (Δϕ_error_) of the output beam through a conventional ϕ-TNLC cell can be considerably reduced by the proposed tandem-2ϕ-TNLC. Although the DoLP values of the output beams through a tandem-2ϕ-TNLC cell are nearly equal to 1 even when the Δϕ_error_ is ignored, the Δϕ_error_, dependent on the wavelength of the incident light, cell gap of the TNLC, and birefringence of the adopted LCs are worth discussion. Regarding the case of the tandem-90°-TNLC cell (Fig. 7(b)), for example, the polarization rotation angles of LP incident light with wavelength of 611 nm, which satisfies the Gooch–Terry condition, at different β angles are the same (90°); those of LP incident light with wavelength of 628 nm, which does not satisfy the Gooch–Terry condition, at β angles of 0°, 22.5°, 30°, and 45° are 86.92°, 87.8°, 87.83°, and 87.88°, respectively. However, according to Eq. (9), the tiny weakness can be further improved by the following approaches: (i) increasing the cell gap and (ii) adopting LCs with high birefringence to approach the criterion of the Mauguin’s condition.

Finally, the tolerance of the cell gap differences between the two conventional ϕ-TNLC cells for assembling the tandem-2ϕ-TNLC polarization rotator with good performance is also discussed. Undoubtedly, the maximum tolerances for different wavelengths in tandem-2ϕ-TNLC polarization rotators having different parameters, such as cell gaps of LC cells, twisted angles, β angles, and birefringence of LCs, are definitely different; therefore, the detailed discussion involves numerous variables. Herein, regarding the case of the tandem-90°-TNLC polarization rotator, whose conditions are consistent with those in the cases shown in Figs 4(a), 5(a), 6(a) and 7(b), two wavelengths (450 and 632.8 nm) of the LP incident lights that do not satisfy the Gooch–Terry condition for the tandem-90°-TNLC cell (cell gap: 20 μm) are selected for theoretical comparison. Additionally, several cell gap differences ranging between 200 nm and 1 μm are selected for theoretical analysis. Just for this case, to obtain the output polarized light whose DoLP value is higher than 0.9 after passing through the tandem-90°-TNLC polarization rotator, the maximum tolerances of the cell gap difference for wavelengths of 450 and 632.8 nm between the two 45°-TNLC cells should be less than approximately 230 and 330 nm, respectively. As previously described, we cannot generally conclude that the maximum tolerance of the cell gap differences for short wavelengths is certainly smaller than that for long wavelengths because the tolerance of the cell gap differences depends on numerous variables.

### Experimental results

In this section, the experimental transmittance, polarization state (χ), and polarization rotation angle error (Δϕ_error_) of the output beam versus the β angles of the LP incident light passing through the conventional 90°-TNLC cell and the tandem-90°-TNLC cell are compared. The probe beam was a He–Ne laser with a wavelength of 632.8 nm. With regard to the prepared conventional 90°-TNLC cell filled with nematic LC (E7), the precise cell gap was approximately 19.251 μm, which satisfied the Mauguin’s condition but not the Gooch–Tarry condition. Moreover, with the combination of the two identical 45°-TNLC cells and consideration of the preparations of the tandem-90°-TNLC cell, the precise cell gaps of the two 45°-TNLC cells filled with nematic LC (E7) were approximately 19.419 and 19.186 μm. The difference in their cell gaps was 233 nm (~1.2%) under the accepted tolerance analyzed by theoretical calculation for 632.8 nm, as shown in the previous section. Notably, the two 45°-TNLC cells satisfied the Mauguin’s condition but not the Gooch–Tarry condition.

**Figure 8:**
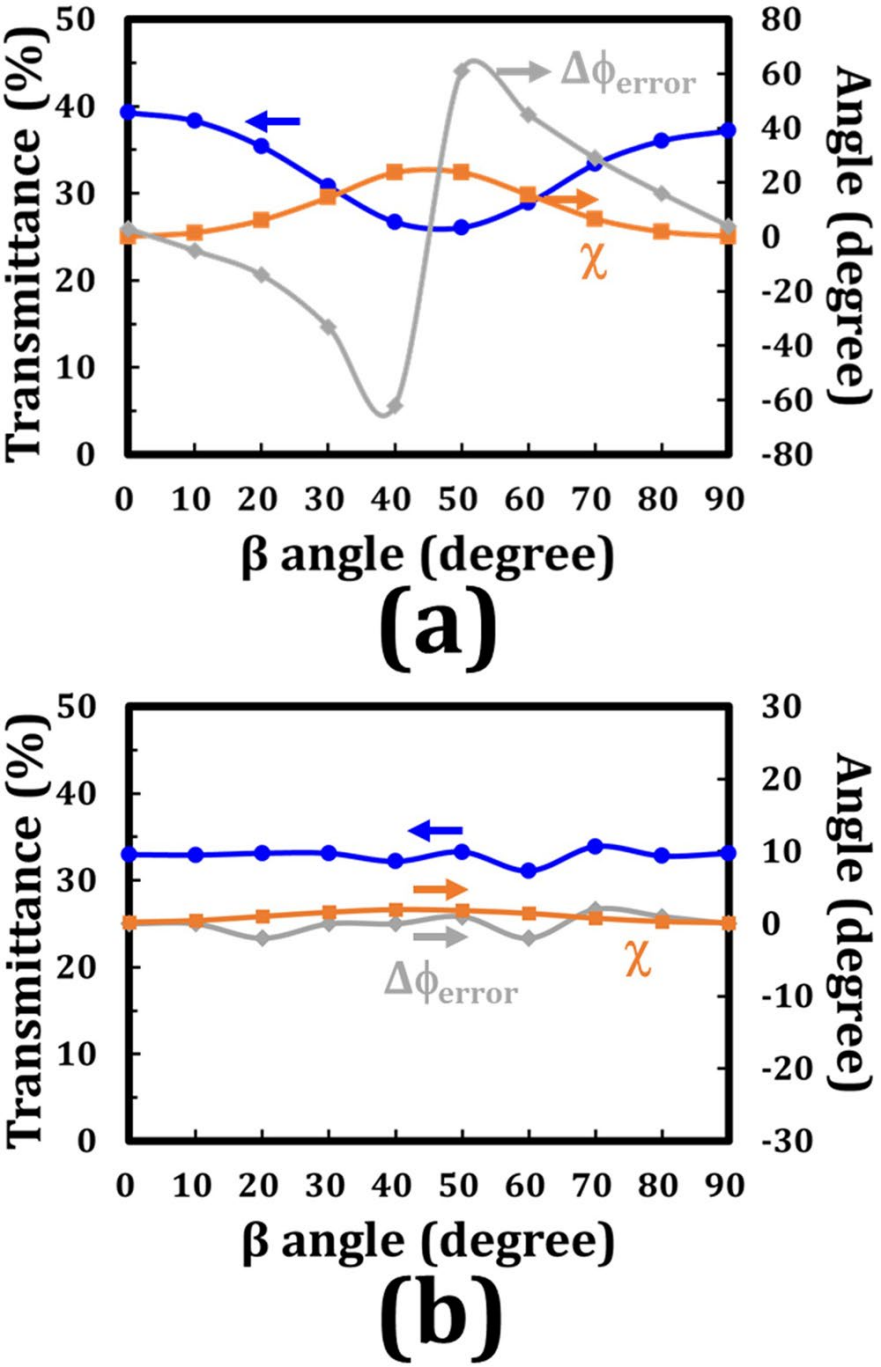
Experimental results of variations in transmittance (blue), χ angle (orange) and Δϕ_error_ angle (gray) as functions of the β angle (0°–90°) of (**a**) a conventional 90°-TNLC cell and (**b**) a tandem-90°-TNLC cell, respectively. Transmittance is defined as the transmittance of the output beam through a TNLC cell and an analyzer with its transmissive axis of 90° with respect to that of the polarizer (β). The light absorbance by polarizers and the light reflection from the boundaries are considered.

According to the experimental results, Fig. 8(a,b) plot the variations in transmittance, χ angle, and Δϕ_error_ angle as functions of the β angle (0°–90°) of the conventional 90°-TNLC cell and the tandem-90°-TNLC cell, respectively. Transmittance is defined as the transmittance of the output beam through a TNLC cell and an analyzer with a transmissive axis of 90° with respect to that of the polarizer (β). The transmission value of 1 is defined as the total power/intensity of the input unpolarized light; therefore, the theoretical maximum of the transmission is 0.5. The transmission was reasonably lower than 0.4 due to the light absorbance by the real polarizers and the reflection/scattering from the boundaries. Considering the conventional 90°-TNLC cell (Fig. 8(a)), the transmittance, χ angle, and Δϕ_error_ angle varied with the β angle because the conventional 90°-TNLC cell was unable to satisfy the Gooch–Tarry condition without the precise calculation and fabrication processes. Hence, the χ angle of approximately 0° was obtained when the β angles were 0° and/or 90°, which satisfied the bisector effect and the theoretical calculation shown in Eq. (5) ^5^. This result suggested that these polarizations of the corresponding output beams were considerably closer to the linear polarization. With exception for the two cases, according to the superposition of the phase retardation resulting from a stack of equally thin plates of birefringent material with small and consecutive rotation of their optical axes, the undesirable phase retardation changed the polarization states of the output beams to be elliptically polarized, thereby increasing their χ and Δϕ_error_ angles. According to the theoretical analyses and experimental results, the polarization directions of the incident beams passing through the tandem-90°-TNLC polarization rotator for the cases of different wavelengths at different β angles cannot be exactly rotated by 90° as previously described. Although small errors of polarization rotation angle exist, the polarization of the output beam is nearly LP. However, regarding the performances of a conventional 90°-TNLC cell, the polarization rotation angle error (Δϕ_error_) and the polarization state (χ) of an output beam for an LP incident light strongly depends on the β angles. Accordingly, the considerable improvements by the proposed tandem-90°-TNLC cell can be clearly examined.

Moreover, regarding the variations in transmittance, χ, and Δϕ_error_ angles as functions of the β angle (0°–90°) in the tandem-90°-TNLC cell (Fig. 8(b)), the obtained experimental results are nearly independent of the polarization plane of the incident light (β angle), which is consistent with those in theoretical analyses. Although the criterion of the Gooch–Tarry condition was absent in the two 45°-TNLC cells, the self-compensation of undesirable phase retardation compensated the polarization of the output beam due to the required arrangement of the two 45°-TNLC cells, as shown in Fig. 1. Accordingly, the TNLC performances can be considerably improved by the tandem approach. Nevertheless, the expected cost of the tandem-TNLC cell is reduction in transmittance, which is mainly caused by the additional boundaries between the substrates and air. Undesirable boundaries can be reduced by a combination of the bottom substrate of the first 45°-TNLC cell with the top substrate of the second 45°-TNLC cell, and/or coating with antireflection films.

**Figure 9:**
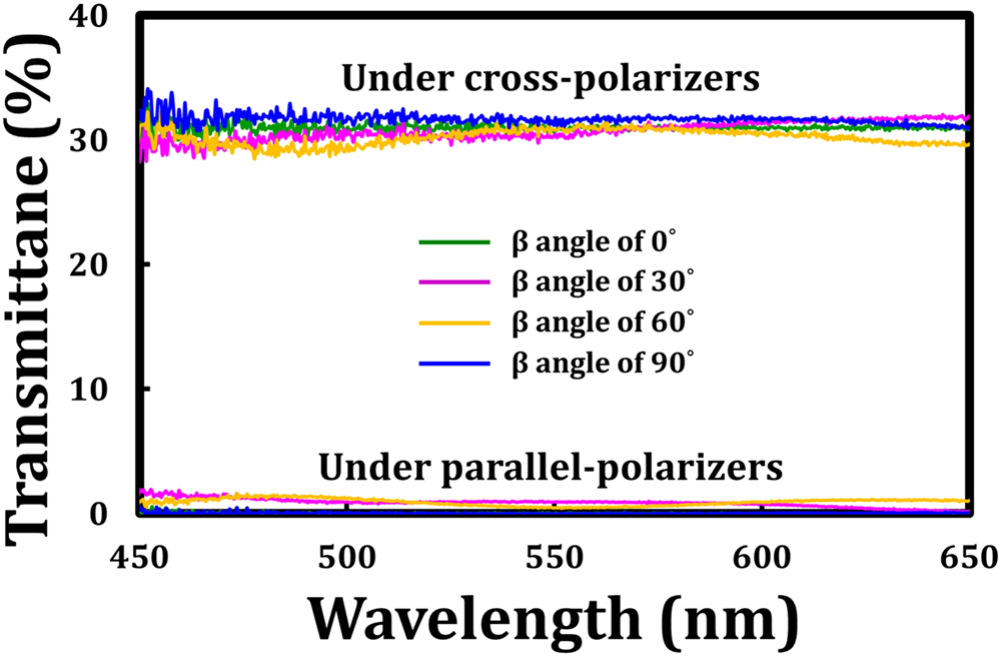
Measured transmission spectra of the tandem-90°-TNLC cell under cross- and parallel-polarizers when the β angles of the incident broadband backlight were 0°, 30°, 60°, and 90°. Cross- and parallel- polarizers represent that the angle made between the transmissive axes of the analyzer (output) and polarizer (input) are 90° and 0°, respectively.

**Figure 10:**
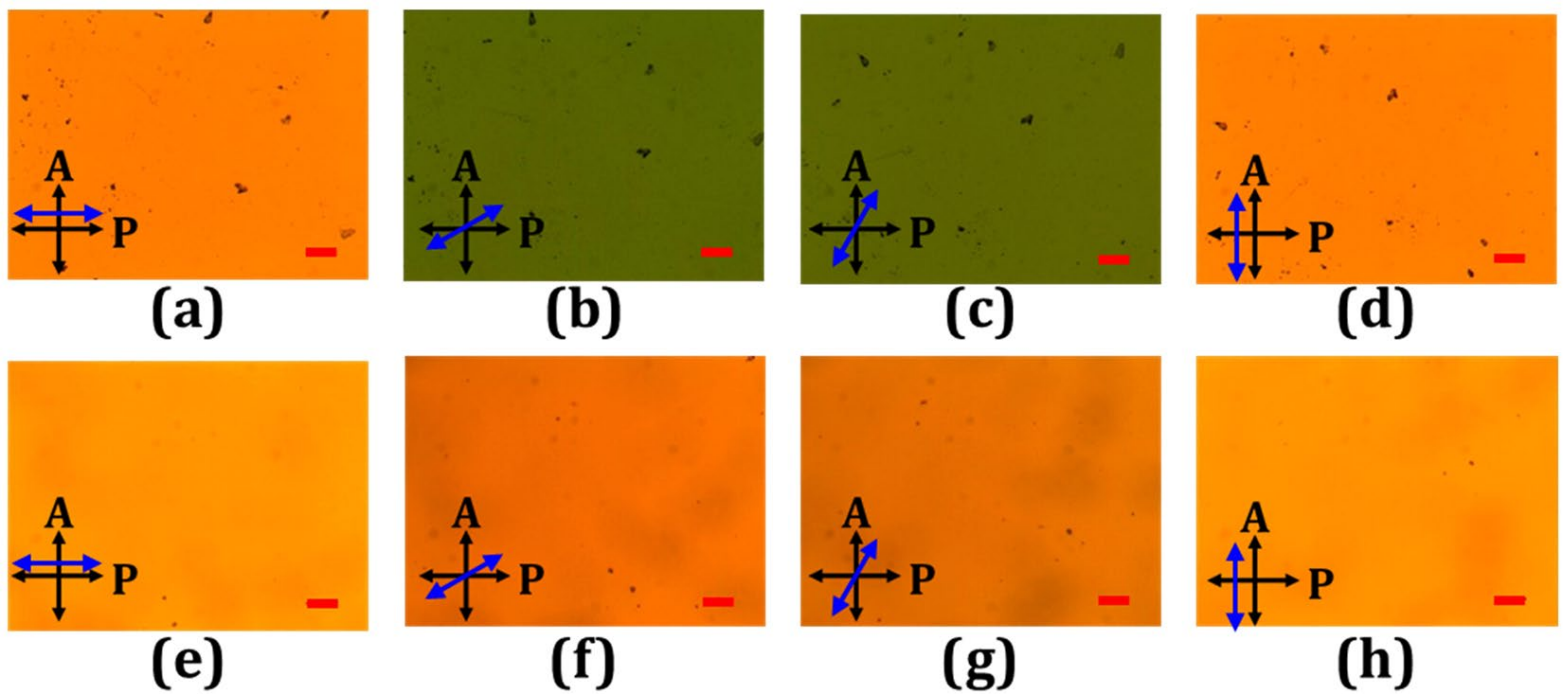
Crossed-polarized POM images of the conventional 90°-TNLC [tandem-90°-TNLC] cell when the β angles of the POM broadband backlight were (**a**) [(**e**)] 0°, (**b**) [(**f**)] 30°, (**c**) [(**g**)] 60°, and (**d**) [(**h**)] 90°. Blue arrow, red scale bar, A, and P are the director of the LCs close to the substrate facing the backlight of the POM, the length of 100 μm, and the transmissive axes of the analyzer and the polarizer, respectively.

The green, purple, yellow, and blue curves in Fig. 9 plot the measured transmission spectra of the tandem-90°-TNLC cell using the same setup in Figs 5(a) and 6(a) when the β angles of the incident broadband backlight were 0°, 30°, 60°, and 90°, respectively. The experimental results on wavelength independence (achromaticity) were also consistent with those of the theoretical analyses shown in Figs 5(a) and 6(a). The experimental results indicated that such a proposed tandem-90°-TNLC cell was an achromatic LC polarization rotator. Finally, Figs 10(a–d) and 10(e-h) present images of the conventional 90°-TNLC and tandem-90°-TNLC cells rotated at β angles of 0°, 30°, 60°, and 90°, respectively, observed under a crossed-polarized optical microscope (POM) with a broadband backlight. Clearly, with the rotations of different β angles, the transmissions of the POM broadband backlight through the conventional 90°-TNLC cell were wavelength-dependent while those through the tandem-90°-TNLC cell were wavelength-independent (achromatic). Notably, the brightness of the cases of 30° and 60° was lower than that of the cases of 0° and 90° because the χ angles of the output polarized light at β angles of 30° and 60° were larger than those of the output polarized light at β angles of 0° and 90°. The transmittance of the output beam through the analyzer was reduced due to the imperfect LP light. Hence, these experimental results were consistent with those shown in Figs 8 and 9 and the theoretical analyses presented in Figs 3(a–d) and 6(a). Therefore, the proposed linear polarization rotator based on a tandem-2ϕ-TNLC cell is an achromatic LC device.

## Conclusion

This paper reports an achromatic linear polarization rotator that is based on tandem-2ϕ-TNLCs comprising two conventional ϕ-TNLC cells. This achromatic linear polarization rotator is wavelength-independent (achromatic) and insensitive to the polarization plane of the incident light. Additionally, the polarization rotation angle error, defined as the angle between the expected polarization direction and the major-axis direction of the output elliptically polarized light, can be considerably reduced. Theoretical analyses by Jones Calculus and 1D-DIMOS confirmed the performances of the polarization rotator. Given the specifically required arrangement in which the LC director close to the last layer of the first ϕ-TNLC cell should be perpendicular to that close to the first layer of the second ϕ-TNLC cell (Fig. 1), the limited performances of the TNLCs based on the tandem-2ϕ-TNLC cell can be nearly eliminated by the self-compensation of the undesirable phase retardation. With the combination of two conventional 45°-TNLC cells and two conventional 22.5°-TNLC cells, appropriate arrangements, and proper applications of external voltage(s), the linear polarization of output LP light with polarization rotations of 0°, ±45°, and 90° can be electrically switched. Hence, such achromatic linear polarization rotators can be potentially used for practical applications.

## Methods

### Material and Cell Preparations

Two pieces of 45°-TNLC cells and one 90°-TNLC cell were prepared to demonstrate the polarization rotation and compare their performances. A nematic LC, E7 (Merck) with a positive dielectric anisotropic constant (ε_∥_ = 19.0; ε_⊥_ = 5.2; Δε = 13.8 for 1 KHz@20 °C), was used as the LC host in all the TNLC cells. The ordinary and extraordinary refractive indexes of E7 were n_o_ = 1.519 and n_e_ = 1.73, respectively, at 20 °C and λ of 632.8 nm. The clearing temperature (T_C_) of E7 was approximately 61 °C. With regard to the empty cells, two indium tin oxide-coated glass substrates were coated with polyimide (PI) layers and rubbed to obtain planar alignments. Each empty cell with 20 μm-thick cell gap was fabricated by the two preceding substrates with rubbed PI layers according to the required twisted angles. The precise cell gaps of the fabricated empty cells were measured by interference with multiple beams^4^. Afterward, the adopted LC (E7) was filled into the empty cells by capillary action. Finally, the fabrication processes of the 45°- and 90°-TNLC cells were completed. The transmittance under cross- and parallel-polarizers, the polarization rotation angle, and the polarization state of the output beams through the conventional 90° TNLC cells and the tandem-90°-TNLCs were measured experimentally.
